# Poly[[μ_2_-1,4-bis­(4,5-dihydro-1,3-oxazol-2-yl)benzene-κ^2^ *N*:*N*′]di-μ_2_-chlorido-cadmium]

**DOI:** 10.1107/S1600536811027036

**Published:** 2011-07-13

**Authors:** Pin-Ning Wang, Chun-Wei Yeh, Hsun-Tsing Lee, Maw-Cherng Suen

**Affiliations:** a Department of Material and Fiber, Nanya Institute of Technology, Chung-Li 320, Taiwan; b Department of Chemistry, Chung-Yuan Christian University, Chung-Li 320, Taiwan; c Department of Materials Science and Engineering, Vanung University, Chung-Li 320, Taiwan

## Abstract

In the title coordination polymer, [CdCl_2_(C_12_H_12_N_2_O_2_)]_*n*_, the Cd^II^ ion, situated on an inversion center, is coordinated by four bridging Cl atoms and two N atoms from two 1,4-bis­(4,5-dihydro-1,3-­oxazol-2-yl)benzene (*L*) ligands in a distorted octa­hedral geometry. Each *L* ligand also lies across an inversion center and bridges two Cd^II^ ions, forming infinite two-dimensional recta­ngular layers running parallel to (010).

## Related literature

For background to coordination polymers with organic ligands, see: Kitagawa *et al.* (2004 ▶); Chiang *et al.* (2008 ▶); Yeh *et al.* (2008 ▶, 2009 ▶); Hsu *et al.* (2009 ▶). For Cd^II^ coordination polymers, see Suen *et al.* (2007*a* ▶,*b* ▶). For related structures, see: Wang *et al.* (2008 ▶).
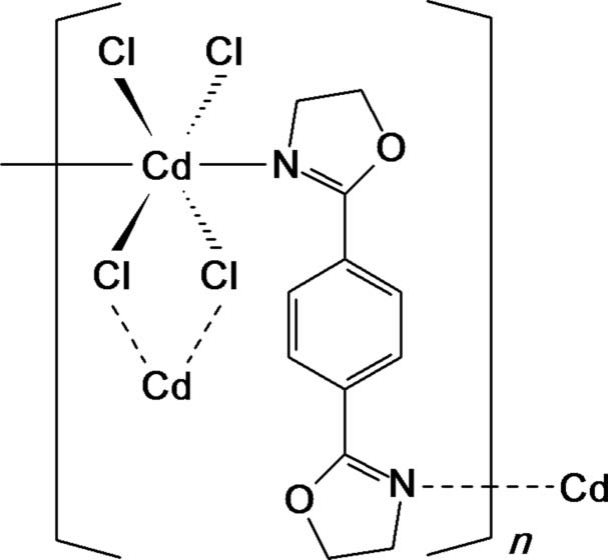


## Experimental

### 

#### Crystal data

[CdCl_2_(C_12_H_12_N_2_O_2_)]
*M* *_r_* = 399.54
Triclinic, 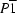

*a* = 3.9242 (4) Å
*b* = 8.0290 (8) Å
*c* = 10.0778 (10) Å
α = 84.632 (2)°
β = 81.458 (2)°
γ = 84.002 (2)°
*V* = 311.30 (5) Å^3^
*Z* = 1
Mo *K*α radiation
μ = 2.18 mm^−1^
*T* = 297 K
0.50 × 0.50 × 0.07 mm

#### Data collection

Bruker SMART CCD area-detector diffractometer
Absorption correction: multi-scan (*SADABS*; Bruker, 1997 ▶) *T*_min_ = 0.319, *T*_max_ = 0.862
1779 measured reflections
1209 independent reflections
1204 reflections with *I* > 2σ(*I*)
*R*_int_ = 0.029

#### Refinement

*R*[*F* ^2^ > 2σ(*F* ^2^)] = 0.044
*wR*(*F* ^2^) = 0.125
*S* = 1.13
1209 reflections
88 parameters
H-atom parameters constrained
Δρ_max_ = 0.93 e Å^−3^
Δρ_min_ = −1.80 e Å^−3^

### 

Data collection: *SMART* (Bruker, 1997 ▶); cell refinement: *SAINT* (Bruker, 1997 ▶); data reduction: *SAINT*; program(s) used to solve structure: *SHELXS97* (Sheldrick, 2008 ▶); program(s) used to refine structure: *SHELXL97* (Sheldrick, 2008 ▶); molecular graphics: DAIMOND (Brandenburg, 2009 ▶); software used to prepare material for publication: *SHELXTL* (Sheldrick, 2008 ▶).

## Supplementary Material

Crystal structure: contains datablock(s) I, global. DOI: 10.1107/S1600536811027036/gw2103sup1.cif

Structure factors: contains datablock(s) I. DOI: 10.1107/S1600536811027036/gw2103Isup2.hkl

Additional supplementary materials:  crystallographic information; 3D view; checkCIF report

## Figures and Tables

**Fig. 1.**
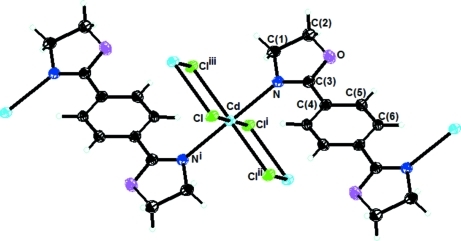
A portion of the two-dimensional net. Ellipsoids are drawn at 30% probability level, and H atoms of spheres of arbitrary radius. Symmetry codes: (i) 1 - x, 1 - y, 1 - z; (ii) x - 1, y, z.

**Fig. 2.**
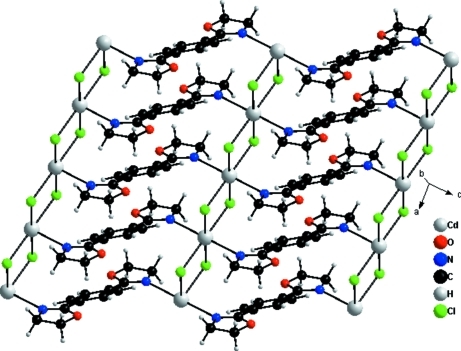
A drawing of the two-dimensional rectangular net.

## supplementary crystallographic information

### Comment

The synthesis of metal coordination polymers has been a subject of intense research due to their interesting structural chemistry and potential applications in gas storage, separation, catalysis, magnetism, luminescence, and drug delivery (Kitagawa *et al.*, 2004). Roles of anion, solvent and ligand comformations in self-assembly of coordination complexes containing polydentate nitrogen ligands are very intersting (Chiang *et al.*, 2008; Yeh *et al.*, 2008; Hsu *et al.*, 2009; Yeh *et al.*, 2009). The Cd^II^ complexes containing polydentate ligands showing various type frameworks are also reported (Suen *et al.*, 2007*a*,b). The Ag(I) complexes containing 1,4-bis(4,5-dihydro-2-oxazolyl)benzene (*L*) ligands has been reported, which show various two-dimensional networks (Wang *et al.*, 2008). The Cd^2+^ cations are sixcoordinated with four Cl atoms and two N atoms from two *L* ligands (Fig. 1). The Cd···Cd distances separated by the bridging *L* ligands and Cl atoms are 10.257 (1) and 3.924 (1) Å, while the ligands adopt the *anti* conformation in the structure (Fig. 2).

### Experimental

An aqueous solution (5.0 ml) of cadmium chloride (1.0 mmol) was layered carefully over a methanolic solution (5.0 ml) of 1,4-bis(4,5-dihydro-2-oxazolyl)benzene (1.0 mmol) in a tube. Colourless crystals were obtained after several weeks. These were washed with methanol and collected in 65.2% yield.

### Refinement

H atoms were constrained to ideal geometries, with C—H = 0.93 (phenyl) or 0.97 (methylene) Å and *U*_iso_(H) = 1.2*U*_eq_(C).

### Crystal data

**Table:**

[CdCl_2_(C_12_H_12_N_2_O_2_)]	*Z* = 1
*M**_r_* = 399.54	*F*(000) = 196
Triclinic, *P*1	*D*_x_ = 2.131 Mg m^−^^3^
Hall symbol: -P 1	Mo *K*α radiation, λ = 0.71073 Å
*a* = 3.9242 (4) Å	Cell parameters from 1711 reflections
*b* = 8.0290 (8) Å	θ = 2.6–26.0°
*c* = 10.0778 (10) Å	µ = 2.18 mm^−^^1^
α = 84.632 (2)°	*T* = 297 K
β = 81.458 (2)°	Parallelepiped, colourless
γ = 84.002 (2)°	0.50 × 0.50 × 0.07 mm
*V* = 311.30 (5) Å^3^	

### Data collection

**Table:**

Bruker SMART CCD area-detector diffractometer	1209 independent reflections
Radiation source: fine-focus sealed tube	1204 reflections with *I* > 2σ(*I*)
graphite	*R*_int_ = 0.029
phi and ω scans	θ_max_ = 26.0°, θ_min_ = 2.1°
Absorption correction: multi-scan (*SADABS*; Bruker, 1997)	*h* = −2→4
*T*_min_ = 0.319, *T*_max_ = 0.862	*k* = −9→9
1779 measured reflections	*l* = −12→12

### Refinement

**Table:**

Refinement on *F*^2^	Primary atom site location: structure-invariant direct methods
Least-squares matrix: full	Secondary atom site location: difference Fourier map
*R*[*F*^2^ > 2σ(*F*^2^)] = 0.044	Hydrogen site location: inferred from neighbouring sites
*wR*(*F*^2^) = 0.125	H-atom parameters constrained
*S* = 1.13	*w* = 1/[σ^2^(*F*_o_^2^) + (0.110*P*)^2^] where *P* = (*F*_o_^2^ + 2*F*_c_^2^)/3
1209 reflections	(Δ/σ)_max_ = 0.001
88 parameters	Δρ_max_ = 0.93 e Å^−^^3^
0 restraints	Δρ_min_ = −1.80 e Å^−^^3^

### Special details

**Table:**

Geometry. All e.s.d.'s (except the e.s.d. in the dihedral angle between two l.s. planes) are estimated using the full covariance matrix. The cell e.s.d.'s are taken into account individually in the estimation of e.s.d.'s in distances, angles and torsion angles; correlations between e.s.d.'s in cell parameters are only used when they are defined by crystal symmetry. An approximate (isotropic) treatment of cell e.s.d.'s is used for estimating e.s.d.'s involving l.s. planes.
Refinement. Refinement of *F*^2^ against ALL reflections. The weighted *R*-factor *wR* and goodness of fit *S* are based on *F*^2^, conventional *R*-factors *R* are based on *F*, with *F* set to zero for negative *F*^2^. The threshold expression of *F*^2^ > σ(*F*^2^) is used only for calculating *R*-factors(gt) *etc*. and is not relevant to the choice of reflections for refinement. *R*-factors based on *F*^2^ are statistically about twice as large as those based on *F*, and *R*- factors based on ALL data will be even larger.

### Fractional atomic coordinates and isotropic or equivalent isotropic displacement parameters (Å^2^)

**Table:**

	*x*	*y*	*z*	*U*_iso_*/*U*_eq_	
Cd	0.5000	0.5000	0.5000	0.0206 (2)	
Cl	0.9164 (2)	0.67051 (12)	0.60009 (9)	0.0232 (3)	
O	0.2884 (9)	0.1013 (4)	0.8636 (3)	0.0366 (7)	
N	0.4705 (9)	0.2869 (4)	0.6933 (3)	0.0214 (6)	
C1	0.6062 (11)	0.1199 (4)	0.6473 (4)	0.0276 (8)	
H1A	0.8569	0.1106	0.6290	0.033*	
H1B	0.5150	0.1001	0.5663	0.033*	
C2	0.4811 (12)	−0.0049 (5)	0.7647 (4)	0.0319 (9)	
H2A	0.3342	−0.0813	0.7368	0.038*	
H2B	0.6751	−0.0698	0.8001	0.038*	
C3	0.3089 (9)	0.2623 (4)	0.8112 (4)	0.0226 (7)	
C4	0.1447 (9)	0.3868 (4)	0.9047 (3)	0.0207 (7)	
C5	0.2302 (9)	0.5530 (4)	0.8878 (3)	0.0217 (7)	
H5A	0.3857	0.5879	0.8141	0.026*	
C6	0.0839 (9)	0.6652 (4)	0.9805 (3)	0.0217 (7)	
H6A	0.1363	0.7764	0.9673	0.026*	

### Atomic displacement parameters (Å^2^)

**Table:**

	*U*^11^	*U*^22^	*U*^33^	*U*^12^	*U*^13^	*U*^23^
Cd	0.0178 (3)	0.0218 (3)	0.0219 (3)	0.00137 (19)	−0.00280 (19)	−0.00334 (19)
Cl	0.0209 (5)	0.0258 (5)	0.0226 (5)	0.0017 (4)	−0.0016 (4)	−0.0068 (4)
O	0.051 (2)	0.0240 (13)	0.0264 (13)	0.0061 (13)	0.0120 (13)	0.0015 (10)
N	0.0240 (15)	0.0221 (15)	0.0168 (14)	0.0029 (11)	−0.0019 (11)	−0.0025 (11)
C1	0.031 (2)	0.0227 (17)	0.0263 (18)	0.0054 (14)	0.0015 (15)	−0.0046 (14)
C2	0.038 (2)	0.0235 (18)	0.0291 (19)	0.0043 (16)	0.0064 (16)	−0.0031 (15)
C3	0.0192 (17)	0.0231 (17)	0.0253 (17)	−0.0002 (13)	−0.0031 (13)	−0.0015 (12)
C4	0.0200 (17)	0.0240 (16)	0.0176 (15)	0.0024 (13)	−0.0023 (12)	−0.0044 (12)
C5	0.0225 (17)	0.0238 (16)	0.0167 (16)	0.0007 (14)	0.0000 (12)	0.0015 (12)
C6	0.0269 (19)	0.0183 (15)	0.0193 (17)	−0.0018 (13)	−0.0023 (13)	−0.0005 (12)

### Geometric parameters (Å, °)

**Table:**

Cd—N^i^	2.467 (3)	C1—H1A	0.9700
Cd—N	2.467 (3)	C1—H1B	0.9700
Cd—Cl	2.6035 (10)	C2—H2A	0.9700
Cd—Cl^i^	2.6035 (10)	C2—H2B	0.9700
Cd—Cl^ii^	2.6557 (9)	C3—C4	1.471 (5)
Cd—Cl^iii^	2.6557 (9)	C4—C5	1.398 (5)
Cl—Cd^iv^	2.6557 (9)	C4—C6^v^	1.413 (5)
O—C3	1.355 (4)	C5—C6	1.380 (5)
O—C2	1.447 (4)	C5—H5A	0.9300
N—C3	1.269 (5)	C6—C4^v^	1.413 (5)
N—C1	1.480 (4)	C6—H6A	0.9300
C1—C2	1.534 (5)		
			
N^i^—Cd—N	180.000 (1)	C2—C1—H1A	110.9
N^i^—Cd—Cl	87.04 (8)	N—C1—H1B	110.9
N—Cd—Cl	92.96 (8)	C2—C1—H1B	110.9
N^i^—Cd—Cl^i^	92.96 (8)	H1A—C1—H1B	108.9
N—Cd—Cl^i^	87.04 (8)	O—C2—C1	103.7 (3)
Cl—Cd—Cl^i^	180.000 (1)	O—C2—H2A	111.0
N^i^—Cd—Cl^ii^	87.28 (7)	C1—C2—H2A	111.0
N—Cd—Cl^ii^	92.72 (7)	O—C2—H2B	111.0
Cl—Cd—Cl^ii^	96.51 (3)	C1—C2—H2B	111.0
Cl^i^—Cd—Cl^ii^	83.49 (3)	H2A—C2—H2B	109.0
N^i^—Cd—Cl^iii^	92.72 (7)	N—C3—O	117.9 (3)
N—Cd—Cl^iii^	87.28 (7)	N—C3—C4	128.7 (3)
Cl—Cd—Cl^iii^	83.49 (3)	O—C3—C4	113.4 (3)
Cl^i^—Cd—Cl^iii^	96.51 (3)	C5—C4—C6^v^	119.2 (3)
Cl^ii^—Cd—Cl^iii^	180.000 (1)	C5—C4—C3	121.3 (3)
Cd—Cl—Cd^iv^	96.51 (3)	C6^v^—C4—C3	119.3 (3)
C3—O—C2	106.9 (3)	C6—C5—C4	120.0 (3)
C3—N—C1	107.0 (3)	C6—C5—H5A	120.0
C3—N—Cd	140.4 (2)	C4—C5—H5A	120.0
C1—N—Cd	109.9 (2)	C5—C6—C4^v^	120.8 (3)
N—C1—C2	104.5 (3)	C5—C6—H6A	119.6
N—C1—H1A	110.9	C4^v^—C6—H6A	119.6

Symmetry codes: (i) −*x*+1, −*y*+1, −*z*+1; (ii) *x*−1, *y*, *z*; (iii) −*x*+2, −*y*+1, −*z*+1; (iv) *x*+1, *y*, *z*; (v) −*x*, −*y*+1, −*z*+2.
